# Pattern and outcome of dog bite injuries among children in Ado-Ekiti, Southwest Nigeria

**DOI:** 10.11604/pamj.2017.27.81.7360

**Published:** 2017-06-02

**Authors:** Ezra Olatunde Ogundare, Oladele Simeon Olatunya, Isaac Oluwadare Oluwayemi, Adekoya Joshua Inubile, Adekunle Bamidele Taiwo, Oyinkansola Tolulope Agaja, Alfred Airemionkhale, Adewale Fabunmi

**Affiliations:** 1 Department of Paediatrics and Child Health, Ekiti State University, Ado-Ekiti, Nigeria; 2 Department of Paediatrics and Child Health, Ekiti State University Teaching Hospital, Ado-Ekiti, Nigeria; 3 Department of Biostatistics, Ekiti State University Teaching Hospital, Ado-Ekiti, Nigeria

**Keywords:** Dog bite, injuries, children, postexposure prophylaxis, rabies

## Abstract

**Introduction:**

Dog bites in humans are a major public health problem. Globally, millions of people are bitten by dogs but most of the fatal cases occur in children. There is paucity of data on dog bite related diseases among Nigerian children. Objectives: to determine the pattern of dog bite injuries and associated health problems among children seen at Ekiti State University Teaching Hospital.

**Methods:**

This is a retrospective study on the clinical data of patients managed for dog bite related injuries between January 2010 and June 2014.

**Results:**

In all, 84 cases of dog bite injuries were managed constituting 0.89% of the total consultations; six (7.1%) had rabies. Most of the victims were aged 6-12 years (60.7%) and majority (71.4%) was boys. Eighty two percent of the victims presented within 24hrs of the injury. Thirty-six (43%) had WHO grade 3 dog bite injury at presentation and the lower limb was the commonest (57.1%) bite site. Use of herbal preparation was the most common pre-hospital treatment 60%. Although 92.9% received anti-rabies vaccine, only 64.3% of them completed the vaccination schedule. The case fatality rate for dog bite was 7.14%. The six that died all presented late, had no post exposure prophylaxis and died within 24 hours of admission.

**Conclusion:**

There is need for public enlightenment on dangers associated with dog bites and also for the government to defray the high cost of post exposure prophylaxis treatment for children.

## Introduction

Dog bite remains a major public health and clinical problem causing palpable fear and anxiety in the patients, the relatives and the attending health care worker not only for the associated morbidity but for the risk of contracting rabies which if not adequately prevented or passes unrecognized almost always result in fatal outcome [1-3]. Dogs are the major reservoir and vector of the rabies virus in Nigeria [4]. Dog bites and rabies are a major public health issue globally and this is not unconnected with the well known fact that rabies is usually fatal once the clinical symptoms develop [5]. Dog bites are the main causes of these preventable traumatic injuries especially in the Paediatric population [6]. Globally, millions of people are bitten by dogs; reports in the USA estimated about 4.5 million dog bites per year and this resulted in 368,265 emergency department visits in 2001 [7]. It was reported that, forty-two percent of dog bite victims are children below fourteen years of age and the injury rate was reported to be highest for children between 5 and 9 years of age with the rate decreasing with increasing age [7]. The injury rate was also reported to be higher among the boys than girls [7].

Dog bites cause a crushing-type wound because of their rounded teeth and strong jaws. Dog bites can result in varying degrees of injuries but fatal injuries occur mostly in children. Victims suffer from psychological and emotional trauma in addition to the physical injuries sustained from the bites [6]. Stray and unvaccinated dogs abound on the streets in Nigeria and other developing countries thereby putting children particularly at risk of either provoked or unprovoked attack by these dogs. Many of these dogs are dogs initially kept as pets, for security purpose, for hunting and also for consumption in some cultures; many of them are later abandoned by the owners. Almost all of them have never had rabies vaccination thus putting victims at risk of rabies [1, 2].

The most feared complication of dog bites is rabies although not all bites result in rabies [8]. It is however essential to assess for and pre-empt the possibility of rabies in every victim of dog bites in Nigeria and most developing countries because of the preponderance of unvaccinated dogs [8]. Rabies is an acute viral disease of the central nervous system that remains largely untreatable. Rabies (meaning *madnesss* in Latin) is caused by rabies virus of the family *Rhabdoviridae* and the genus *Lyssavirus* [9]. The rabies virus affects all warm-blooded animals including man and the outcome is usually fatal [10]. It causes an acute encephalomyelitis and it is typically transmitted from the saliva of a rabid animal via a bite, scratch or mucous membrane exposure [5]. Dogs and cats are the major reservoirs and vectors of rabies transmission especially in Africa and Asia whereas in the Americas, bats are more important in transmitting the disease [11]. Rabies is almost always fatal if post-exposure prophylaxis (PEP) is not administered before the development of symptoms [5]. Globally, about 55,000 human deaths occur annually and over 98 percent of these mortalities are caused by canine rabies [12]. Over 50% of these mortalities occur in children less than 15 years of age [13]. Africa accounts for approximately 24,200 deaths [14]. The only way of preventing rabies is through the administration of pre-exposure immunization to individuals at risk, administration of PEP with or without human rabies immune globulin (HRIG) and control of rabies in animal population [15]. Human diploid cell vaccine in combination with rabies immune globulin when administered promptly to rabies-exposed patients after appropriate wound care is virtually 100% effective in preventing human death [16].

This study was carried out to determine the prevalence and pattern as well as the outcome of dog bite injuries among children seen at the Ekiti State University Teaching Hospital (EKSUTH), Ado-Ekiti, Nigeria.

## Methods

**Study setting**: The study was an observational retrospective study carried out at the children emergency and paediatric out-patient units of Ekiti State University Teaching Hospital (EKSUTH), Ado-Ekiti, Nigeria. The EKSUTH is a tertiary health facility located in Ado Ekiti, the capital of Ekiti State. It receives patients from all parts of the state and other adjoining states like Kogi, Kwara, Osun and Ondo.

**Data collection**: The study reviewed the clinical data of patients managed for dog bite related injuries and rabies over a four and half year period between January 2010 and June 2014. A proforma was designed to extract relevant clinical data from the case records. Information extracted included the age, sex of the victims, site of the bite, time of presentation in the hospital, pre-hospital treatment, hospital treatment including post-exposure prophylaxis and complication. The severity of the injury was determined using the WHO dog bite injury grading system [17].

**Ethical approval**: An approval was obtained from the Ethics and Research Committee of the Ekiti State University Teaching Hospital Ado Ekiti.

**Data analysis**: The information obtained was entered into personal computer and analysed using SPSS version 18. Data was presented in numbers and percentages, chi square was used to compare groups and a p-value of <0.05 was considered significant.

## Results

**Figure 1:**
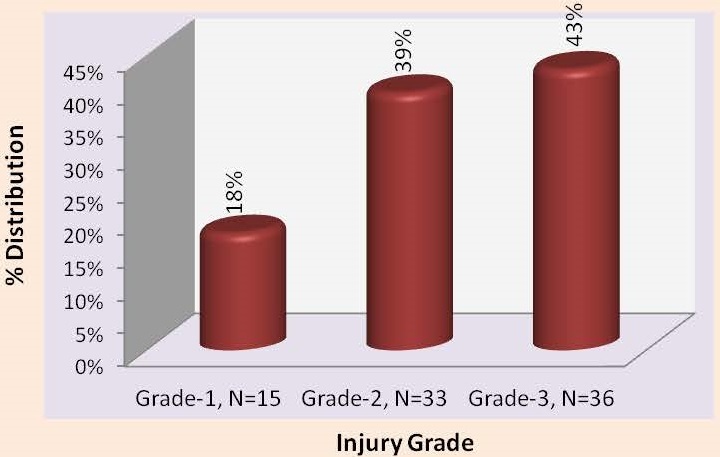
Dog bite wound severity grading in the patients using WHO criteria

**A** total of 9438 patients were seen at the children emergency and paediatric out-patient unit of EKSUTH during the four and half year study period. Eighty-four cases of dog bite related injuries were managed during the period constituting 0.89% of the total consultations and six of the 84 children presented with clinical features of rabies. In most 60 (71.4%) patients, the circumstances surrounding the dog bite was not due to provocation and the dog owners were known in 66 (78.6%) of cases but the vaccination history of the dog was validated in only one (1.2%) of the cases. Majority of the patients 69 (82.1%), presented within 24hours of sustaining the dog bite injury while 9 (10.7%) presented between 24 to 48 hours and the remaining 6 (7.1%) after several weeks. As shown in Table 1, most of the victims are in the age range 6 – 12 years (60.7%), sixty boys (71.4%) were involved giving a male: female ratio of 2.5:1. About 93% of the affected children were from the middle and lower socioeconomic classes. Thirty-three children (39.3%) presented with wounds while 6 (7.14%) victims presented for the first time, in the hospital with central nervous system symptoms weeks after sustaining dog bite injury. The most common pre-hospital treatment received by the victims was the use of herbal preparation in 34 (40.5%), washing bite site with either water or disinfectants in 17 (20.2%) and inscription of scarification marks and tying bite site with ropes to prevent venous drainage in 8 (9.6%) cases. The remaining 25 (29.7%) patients did not receive any pre-hospital treatment. The lower limb was the most common site of bite in about 57% of cases (Table 2). Thirty-six (43.0%) of the victims presented with grade 3 WHO wound severity grading as shown in Figure 1.

**Table 1:** Socio-demographic characteristics and symptoms of dog bite victims

Variable	Frequency	Percentage (%)
**Age range**		
0 – 5 yrs	12	14.3
6 – 12 yrs	51	60.7
> 12 yrs	21	25.0
**Total**	**84**	**100.0**
		
**Sex**		
Male	60	71.4
Female	24	28.6
**Total**	**84**	**100.0**
		
**Social Class**		
Lower	42	50.0
Middle	36	42.9
Upper	6	7.1
**Total**	**84**	**100.0**
		
**Presenting symptom**		
Wounds	69	82.1
Lacerations	9	10.71
Bleeding	18	21.43
Fever	6	7.14
Pain/ Swellings	12	14.29
CNS symptoms	6	7.14

**Table 2:** Sites of dog bite injury

Site of injury	Number of victims	Percentage (%)
Lower limb	48	57.2
Upper limb	24	28.6
Head & Neck	6	7.1
Trunk/ Buttocks	6	7.1
Total	84	100.0

Treatments received in the hospital ranged from washing the bite site with soap and water, to suturing of lacerations and wound dressing, analgesics, tetanus prophylaxis, anti-rabies vaccination (ARV), intravenous fluids and diazepam administration as well as antibiotics administration as shown in Table 3. The treatment administered is determined by the severity or grade of the injury. Patients with higher grades of injury significantly received more hospital treatments (P=0.001) (Table 3). Human rabies immune globulin was not administered on any of the patients.

**Table 3:** Comparison of injury grade with treatment received at the hospital

		Injury grade			Total
		Grade-1	Grade-2	Grade-3	
**Treatment at Hospital**	Anti-Rabies & Tetanus prophylaxis	0	12	18	30
	Anti-Rabies &Wound dressing	6	6	6	18
	Anti-Rabies & Analgesics	0	3	6	9
	Anti-Rabies & Antibiotics	9	9	3	21
	Wound dressing, Antibiotics & Tetanus prophylaxis	0	0	3	3
	Wound dressing, Antibiotics, Tetanus prophylaxis & Analgesics	0	3	0	3
**Total**		**15**	**33**	**36**	**84**

Chi-square value = 33.309 & P-value = 0.001

Although seventy-eight (92.9%) of the victims had post-exposure prophylaxis (PEP) with anti-rabies vaccine (Table 1), only 45 (53.6%) of them were managed successfully and subsequently discharged after ensuring adequate wound healing and completion of the vaccination regimen. Thirty-three (39.3%) were lost to follow up and did not complete the vaccination series, the remaining six victims (7.1%) all, from the lower socioeconomic class who presented late with features of rabies such as hydrophobia, restlessness and aggression died within 24 hours of admission. All the patients who died presented weeks after sustaining dog bite injuries and they did not receive any anti rabies vaccination before presentation thereby giving rabies a case fatality rate of 100% in this study. None among those who had anti-rabies vaccine died (P=0.001) (Table 4). Post mortem examination was not done on any of the victims who died because the parents or care givers declined consent for post mortem examination.

**Table 4:** Outcome of treatment

	OUTCOME				
		Dead	Discharged	Lost to Follow up	Total
ARV Administration	No	6	0	0	6
	Yes	0	45	33	78
Total		6	45	33	84

Chi-square value = 84.000 & P-value = 0.001; NB: ARV-Anti Rabies Vaccine

## Discussion

This study showed that dog bite related injuries are not a common presentation both in the children emergency unit as well as the out-patient department of our hospital as it constituted about 0.89% of the total consultation over the study period. This figure is however higher than 0.31% and 0.24% reported by Schalamon et al [18] and Kahn et al [19] in their studies respectively. This prevalence is however less than 1.5% documented by Dwyer et al [6] at a pediatric trauma unit in South Africa. For the families that lost their wards to dog bite related injuries, it was a 100% loss hence, dog bites still remains a major public health issue in most developing countries because these deaths can be prevented. The high vulnerability of children to dog bite injury as shown by previous studies conducted locally and internationally [1, 7, 20, 21], has been attributed to children’s inability to defend themselves, their small stature and also to the fact that they may provoke the attack in the first instance[1]. Whereas adults are able to stand up to the dogs and defend themselves in most cases, there were many reported cases of unprovoked attacks in this study further highlighting the need to protect children from undue attacks by dogs in the study area. It might be important to come up with prevention programs for children since they are the most vulnerable group as evidenced by this study. Prevention programs such as teaching children how to respond to, behave around and interact with dogs; a randomized controlled trial of a school based intervention in Australia documented a substantial reduction in children’s approach to and interaction with a strange dog [22].

This study revealed that children between the ages of 6 and 12 years are more commonly bitten by dogs and also showed male preponderance (71.4%) among the victims which is similar to findings in previous studies [1, 20] and this is not unconnected with the natural behaviour of boys who are more adventurous and daring than their female counterparts, they are also likely to yell, play with or grab the dogs if possible, which put them at risk of being bitten. The most common site of bites in this study was the lower limb which is similar to what was documented by Abubakar et al [1] in Jos and Iyalohme et al [5] in Ekpoma, Edo state. Similar to these studies [1, 5], head and neck injuries were uncommon in our study. Majority of the bites were from stray dogs whose owners were known to the victims. This is a common practice in our environment as dog owners mostly do not restrain their dogs and they allow them to wander about the streets. Many of these dogs are not well fed by their owners and as such wander around looking for food. No wonder that there was evidence of vaccination in just one (1.2%) of the cases seen in our hospital during the study period. Hence, mass vaccination of domestic dogs as well as removal of stray dogs can help in reducing the risk of dog bites as this has successfully eliminated or controlled domestic dog rabies in many parts of the world [23]. Responsible dog ownership should also be included as part of the preventive measures although this seems not to be feasible with the current economic situation in most homes in the country.

Post exposure administration of human dipoid cell vaccine in combination with human rabies immune globulin soon after bite by suspected rabid dogs or other canines have been found to be very effective in controlling human rabies [16]. The recommended post exposure prophylaxis is intramuscular administration of human diploid cell vaccine into the deltoid on days 0, 3, 7, 14, and 28. Injections can be given into anterolateral thigh for children [24]. Only a little above 50% of the dog bite victims in this study completed the PEP regimen which is slightly lower than the 60.5% documented by Abubakar et al [1] in Jos. This is probably not unconnected with the cost of the vaccine as most of the victims in this study were from the middle and lower socioeconomic classes. A single vial of anti rabies vaccine (ARV) costs two thousand, three hundred and twenty naira ($ 11.7 equivalent) alone asides other adjunct materials needed for its administration. Given the high preponderance of poverty in Nigeria [25], it may be important for the government to look at the possibility of subsidizing the cost of this vaccine. The development of a safe, inexpensive anti-rabies vaccine that can afford adequate protection from a single dose will also go a long way in protecting the huge population of children who are at risk. None of our patient had the human rabies immune globulin. This may also be due to the fact that the attending physicians did not prescribe it alongside the human diploid cell vaccine or that the care givers couldn’t afford it because of the exorbitant cost or probably because it was not available.

Eighty-two percent of the victims in this study presented within 24 hours of injury which is commendable but this is slightly lower than 87.7% reported by Abubakar et al [1] in Jos. Most of our patients had inappropriate home remedies administered before presentation in the hospital and this could have delayed hospital presentation in some of them thus putting the victims at risk of developing complications or even a false sense of assurance when in the real sense the problem was yet to fully evolve. Hence, public enlightenment campaign would be of help in encouraging early presentation as well as informing the public on the right thing to do if they encounter such victims. The six deaths recorded in this study, were from the lower social class, they did not receive PEP with ARV and also presented late with features of rabies and all of them died within 24 hours of presentation. These observations not only underscore the importance of timely PEP in preventing rabies following dog bite injury, but also highlight the unholy alliances between poverty, ignorance, and inadequate access to quality health care in preventing avoidable deaths. The parents of the affected children might have acted out of ignorance or lack of financial power to be able to access care and purchase the human diploid vaccine to complete the PEP regimen among other treatments. There is therefore the need for more aggressive health education of the public and also for the provision of subsidy on the vaccine including the human rabies immune globulin as well as other cares for children with dog bite injury. This will make health care affordable at our health facilities and help to prevent avoidable deaths. One major way of alleviating this burden is the rapid implementation of the newly passed National Health Bill in Nigeria. The bill gives financial leverage to Nigerians on health care costs among other benefits [26].

## Conclusion

In conclusion, this study revealed that dog bite related injuries and rabies are not common reasons for consultation at the children emergency and paediatric outpatient units. However, it highlights the need for public health awareness on the health challenges posed by dog bites because of its high case fatality rate as shown in this study. It also points out the need for public health education on the immediate management of dog bite victims and the preventive measures that need to be put in place to prevent dog bite injuries and also inappropriate pre-hospital management as well as ensuring the availability and affordability of the human diploid cell vaccine and the human rabies immune globulin at our public health facilities. Caregivers should seek medical care in earnest whenever their wards are involved in dog bite injury as delaying may lead to grievous health consequences. **Limitations:** Apart from its retrospective nature, this study may not be the true representation of all the children with dog bite injuries as some may not have presented in the hospital at all or there may be some children who could have presented to other health care facilities or even died hence, the low sample yield. Nonetheless, most children with injuries are usually taken to nearby hospitals and the study centre being the only tertiary facility in the state capital is likely to receive the bulk of the patients. Moreover, in the resource poor settings of Nigeria, hospital based studies are mostly sources of biomedical research data.

### What is known about this topic

- Dog bites and rabies are important public health issues worldwide;
- Children constitute a vast majority of the victims of dog bites;
- Rabies is still a major cause of mortality following dog bite injuries.

### What this study adds

- The study highlights the need for more awareness on the immediate care for dog bite victims and prevention of dog bite injuries using school based prevention programs for children, mass media and other means;
- The need for the enforcement of regulations for dog licensing and rabies vaccination for the dogs;
- The need for the development of single-dose anti-rabies vaccine to encourage compliance with post-exposure prophylaxis.

## Competing interests

The authors declare no competing interest.
